# Vein of Galen malformation presenting as progressive macrocephaly in a 12-month-old girl: A case report

**DOI:** 10.1177/2050313X251364367

**Published:** 2025-08-07

**Authors:** Lindsey Cortes

**Affiliations:** 1 Department of General Pediatrics, UT Health San Antonio, TX, USA

**Keywords:** cerebral veins, vein of Galen malformations, scalp, megalencephaly, magnetic resonance imaging

## Abstract

Vein of Galen malformations represent about 30% of pediatric vascular abnormalities. Depending on the type, they can present insidiously with macrocephaly in the absence of other symptoms and are often misdiagnosed as benign, especially if not captured by initial imaging. The objective of this case report is to highlight this malformation to avoid incorrect diagnoses leading to a delay in proper treatment. Herein, a 12-month-old female developed progressive macrocephaly without loss of developmental milestones or focal deficits. Initial diagnostic testing with ultrasound revealed benign extra-axial fluid as the etiology at 6 months of age. With the continued progression of her macrocephaly accompanied by prominent scalp veins, magnetic resonance imaging of the brain revealed a dilated vein of Galen. The patient underwent successful cerebral embolization of a mural type vein of Galen malformation with stabilization of her head circumference by 18 months of age. This case demonstrates a clinician’s need to maintain a high index of suspicion and broad differential for children with worsening macrocephaly despite normal neurodevelopment and/or initial imaging. Recognized vein of Galen malformations are infrequently encountered in the pediatric setting as an etiology of a very common finding in infancy, macrocephaly.

## Introduction

Vein of Galen malformations (VOGM) represent about 30% of pediatric vascular abnormalities.^1^ They are congenital vascular malformations comprised of multiple arteriovenous (AV) shunts between choroidal arteries and the embryonic precursor to the vein of Galen, the median prosencephalic vein of Markowski, making the vein of Galen a misnomer.^2^ Depending on the type, they can present insidiously with macrocephaly in the absence of other symptoms and are often misdiagnosed as benign, especially if not captured by initial imaging. Choroidal VOGM are often more severe due to high volume shunting, manifesting prenatally or in the neonatal period as high-output heart failure.^1^ Infants and older children with smaller malformations or those of the mural type present with hydrocephalus, dilated scalp veins, macrocephaly, and/or seizures.^3^ Ultrasound is a quick, noninvasive option for imaging, with angiography being the gold standard. The advent of endovascular embolization as treatment has greatly reduced mortality in the pediatric population. This report illustrates a vein of Galen diagnosis in the setting of macrocephaly but in the absence of other symptoms.^4^

## Case presentation

Twelve-month-old girl presented to her pediatrician’s office for her well-child check. She had a known history of macrocephaly, dating back to 1 month of age. There was no known family history of macrocephaly. Prenatal examination, including routine ultrasounds and noninvasive prenatal tests, revealed normal results. The child was born at 39 weeks via vacuum-assisted vaginal delivery. Resuscitation included tactile stimulation and bulb suction with a good result. Apgars at 1 and 5 min were 8 and 9, respectively. Pregnancy was uncomplicated with negative serologies. Birth weight was 3810 g. She did require a brief sepsis watch for prolonged rupture of membranes, but workup was found to be negative. She passed her critical congenital heart disease screener. Her total bilirubin at 28 h of life was noted to be high intermediate risk at 8.4. Subsequent recheck at 44 h of life showed a level of 12.7, which was noted to be high risk, leading to initiation of phototherapy for 24 h. Bilirubin appropriately downtrended to low risk at 63 h of life to 10.0.

Her head circumference at 7 days of life was noted to be at the 82nd%, 35.6 cm. Her weight at that time was 3629 g (57th%) as she was not yet back at birth weight, and length was 53.3 cm (95th%). Head circumference uptrended to the 97th% by 1 month of life (39 cm), weight was at the 60th% (4479 g), and length was at 80th% (55.9 cm). By 2 months of age, head circumference was stable at the 97th% with a measurement of 40.6 cm. Length and height continued to track appropriately. At that visit, she was noticed to have a 4 cm-sized boggy area to her left parietal skull near the apex without overlying skin changes. There was no concern for fracture or recent trauma, but given the history of vacuum-assisted delivery, it was presumed to be a benign subaponeurotic fluid collection.

She returned at 4 months of age with continued superficial scalp swelling, stable in size, and nontender to palpation. By this time, her head circumference was now at the 99th% (43.8 cm). Weight was at the 47th% (6520 g) and length at the 93rd% (66 cm). During all visits, she remained afebrile and hemodynamically stable. She had good pulses and perfusion without any evidence of murmurs, rubs, or gallops. The infant was otherwise developing normally with an intact neurological exam. The remainder of her exam findings were appropriate for her age. Given the stability of the fluid collection, a decision was made to monitor until her next well-child exam, and if no improvement at that time, to perform imaging.

Infant returned at 6 months of age with obvious macrocephaly and no change in the fluid collection on her scalp. Her head circumference jumped to 46.4 cm, >99th%. Weight was at the 57th% (7541 g) and length at the 80th% (67.9 cm). There were no reports of emesis, irritability, or lethargy. All vitals remained within normal limits, and her cardiac and neurological exams remained appropriate. Echoencephalography was ordered and obtained, given anterior fontanelle was still open to rule out hydrocephalus. Head ultrasound, without Doppler, demonstrated mild prominence of interhemispheric and convexity extra-axial fluid spaces that was deemed to be common in the first 18 months of life, but no hydrocephalus. Given this information, follow-up was made for her 9-month well check.

At 9 months of age, her head circumference remained >99th% with a measurement of 48.3 cm. Weight and length were trending along prior parameters. She continued to eat and drink normally. An Ages and Stages questionnaire was done given her age, which showed her passing in all categories with the exception of gross motor, which was borderline. She remained intact neurologically with an open and flat fontanelle, no evidence of skull asymmetry, or frontal bossing. She did begin to manifest prominent veins on her scalp. With her continued normal exam and reassuring head ultrasound, it was decided to wait until her 12-month check-up for further imaging if her head circumference continued to uptrend at such a rapid pace.

**Figure 1.**
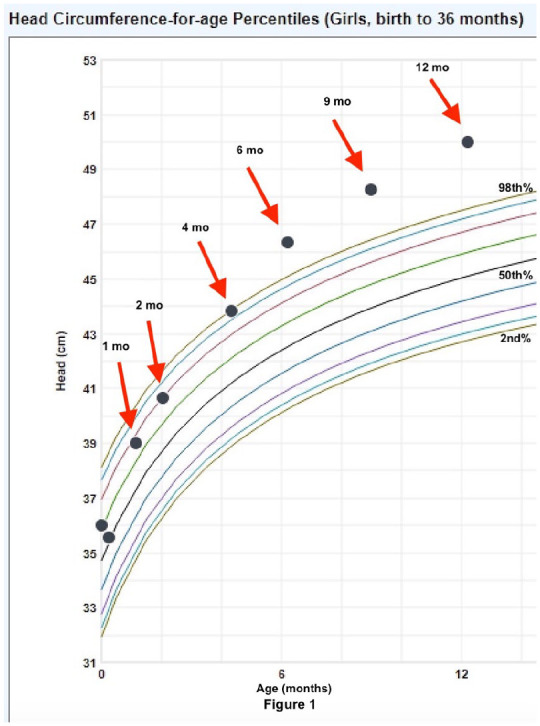
WHO head circumference-for-age chart of her occipitofrontal circumference progression.

At her 12-month visit, her head circumference had increased to 50 cm without plateau (Figure 1). Weight-for-age was at the 64th%, height-for-age was at the 41st%, both trending appropriately. Temperature was 98.8° Fahrenheit, heart rate was 127, respiratory rate was 28, and she was sating 97% on room air. She remained asymptomatic with a normal cardiac and neurological exam and an open anterior fontanelle without bulging. Magnetic resonance imaging (MRI) with and without contrast was ordered to evaluate for other etiologies that could be contributing to her macrocephaly.

**Figure 2.**
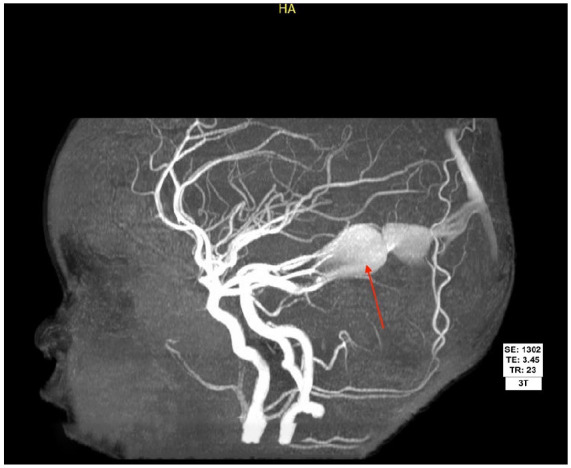
Brain magnetic resonance angiogram with MIP showing dilated left posterior cerebral artery supplying the aneurysmal dilated midline venous pouch representing vein of Galen malformation, labeled with red arrow. MIP: maximum intensity projection.

MRI with and without contrast showed a high-flow AV fistula between the P2 segment of the left posterior cerebral artery and a markedly dilated distal left basal vein of Rosenthal, which subsequently flows into a dilated vein of Galen, straight sinus, and persistent falcine sinus. There were also numerous enlarged posterior scalp vessels noted, representing venous structures due to communication of the superior sagittal sinus with the scalp veins via a transdiploic venous channel. There was no evidence of intracranial hemorrhage, acute infarct, or hydrocephalus. Due to these findings, magnetic resonance angiogram head with and without contrast was also obtained, confirming the above findings (Figure 2).

Neurosurgery was immediately consulted. She was scheduled the following day in the outpatient setting for evaluation. She was presumed to have a VOGM based on imaging. She underwent successful cerebral embolization by an interventional neuroradiologist with over 50 years of experience via transarterial approach using 25% n-butyl-cyanoacrylate (n-BCA), with her ultimate diagnosis as VOGM, mural type with a single fistula supplied from the left posterior medial choroidal artery (Figure 2). Her superficial scalp swelling was actually suspected to be a subcutaneous deep hemangioma with sinus pericranii, an abnormal connection between intracranial and extracranial veins. She tolerated the procedure well, with near complete resolution of vein of Galen perfusion during left vertebral angiogram post-n-BCA. She had eventual stabilization of her head circumference by her 18-month checkup and a dramatic increase in her developmental skills, with a normal angiogram 5 months post-op.

## Discussion

Macrocephaly is defined as a head circumference >97th% or two standard deviations above the mean.^5^ The differential diagnosis for macrocephaly in infants and children is broad. A familial etiology for her macrocephaly is considered, but less likely given continued acceleration of head growth rather than stabilization or slowing after 6 months of age.^5^ Other etiologies include hydrocephalus (communicating or noncommunicating), benign extra-axial fluid of infancy, intracranial hemorrhage, AV malformations (AVMs), space-occupying lesions (benign or malignant), infection, brain malformations, skeletal dysplasias of the cranium, and metabolic central nervous system disease. Many of the above etiologies are unlikely given she had no focal neurological deficits, dysmorphic features, developmental delay, or evidence of increased intracranial pressure (vomiting, seizures, altered mental status, and irritability). Despite benign extra-axial fluid of infancy being the working diagnosis with presence on head ultrasound, absence of hydrocephalus, and overall normal neurodevelopment, strong suspicion remained for an alternative diagnosis, such as an AVM, given the degree of head growth velocity over time.

VOGM represent about 30% of pediatric vascular malformations.^1^ These congenital aneurysmal formations within the brain develop between 6 and 11 weeks of gestation and are comprised of multiple AV shunts between choroidal arteries and a midline venous sac, which gradually enlarges, known as the median prosencephalic vein of Markowski.^3^ This embryonic vein would otherwise normally involute and become the vein of Galen with normal anatomy.^3^ There are two types: choroidal and mural. The choroidal type is often more severe and manifests earlier—either prenatally or soon after birth—due to high volume shunting between all choroidal arteries plus their feeders and the venous aneurysm.^1^ The mural type clinically manifests later, secondary to the formation of AV fistula(s) within the aneurysmal wall of the vein of Markowski.^6^

The typical manifestation in neonates is heart failure.^7^ Symptoms include poor feeding, tachypnea, color change, and cardiomegaly. The flow through the VOGM increases after birth without competition from the low resistance placental circulation, leading to increased venous return to the right atrium and ultimately pulmonary hypertension.^6^ Myocardial ischemia can also ensue secondary to decreased coronary artery blood flow aside from the ischemia that can occur to the cerebral parenchyma from blood steal towards the VOGM.^8^ Outside of the newborn period, infants can manifest with hydrocephalus, macrocephaly, seizures, dilated scalp veins, cranial bruit, developmental delay, and/or proptosis.^3^ Hydrocephalus is suspected to be secondary to direct compression of the aqueduct of Sylvius by the venous aneurysm, in addition to decreased cerebrospinal fluid reabsorption from elevated intracranial venous pressure.^2^ As for older children and adults, VOGMs are often small with limited shunting, but headache is the primary presenting symptom that can be due to hemorrhage, either subarachnoid or intraparenchymal.^2^ Despite VOGMs of the mural type manifesting later, her case was unique, given that her only symptom initially was macrocephaly until 9 months of age.

Ideally, diagnosis of VOGMs is made prenatally with a detailed level II ultrasound in the second trimester to prevent subsequent complications. However, diagnosis is usually made in the third trimester when adequate dilatation can be detected.^9^ Turbulent blood flow within the lesion serves as a characteristic feature on color Doppler after initial findings of an enlarged, midline fluid-filled mass with low echogenicity.^10^ Fetal MRI is considered superior for confirmation of the extent of the malformation along with its vascular structure, which can be done around 32–34 weeks gestation.^10^ In this case, there was no mention to the family regarding abnormalities on prenatal ultrasounds, including the anatomy scan, leading to a delayed diagnosis from the start.

Imaging options postnatally include ultrasound, computed tomography (CT), MRI, and angiography. Head ultrasound, which can also include Doppler, is often an easily accessible method to view cerebral ventricles, midline structures, and brain parenchyma without the need for sedation, but does depend on the experience of the sonographer and/or radiologist.^3^ Positive results can be missed in the setting of inexperience. In terms of CT use, CT angiography is preferred as it remains a minimally invasive modality while offering detailed vasculature via its high spatial resolution, with minimal to no sedation required.^2^ However, this method necessitates the use of radiation and the administration of contrast, both of which are essential for accurate imaging. MRI is able to offer more detail regarding the size of the VOGM, quantity of arterial feeders, and ventricular system abnormalities such as cerebral atrophy or aqueduct compression.^1^ Angiography, however, is considered the gold standard. It offers precise detail of the VOGM angioarchitecture while allowing endovascular access and management either via the umbilical artery in the neonatal period or femoral artery (recommended around the 4th–6th month of life if the infant is stable to allow for brain maturation and maximum efficacy).^1^ While ultrasound was used as the initial imaging modality in this case, Doppler was not an available option at our institution, further delaying the diagnosis. Mere observation based on benign extra-axial fluid findings could have led to devastating consequences.

Endovascular treatment, as mentioned above, is the preferred modality for management of VOGMs. Prognosis in neonates with VOGMs previously, in terms of fatality rates, was near 100% prior to the advent of endovascular therapy.^8^ Diuretics, inotropes, and other agents can be used to stabilize neonates with heart failure prior to embolization.^1^ The ultimate goal is to occlude off any and all fistulas to restore normal circulation to the brain, which can occur in stages.^6^ Endovascular options include transarterial and transvenous techniques.^2^ Risks include vascular injury to vessels, groin hematoma, intracranial hemorrhage, and stroke. n-BCA is the glue often selected for use in the transarterial approach to embolize the fistula.^7^ In a transvenous approach, the femoral or jugular vein is accessed with multiple coils and then deployed into the venous portion of the malformation.^2^ However, this carries the risk of infarction or perforation of the venous aneurysm.^7^ In those patients with hydrocephalus, ventriculoperitoneal shunts are often not needed post-embolization due to the decline in venous pressure and reduction in size of the dilated vein.^6^ Neurological outcomes have drastically improved as a result of endovascular embolization.

## Conclusion

VOGM are infrequently encountered in the pediatric setting. Given the lack of detection on prenatal or postnatal ultrasound without Doppler, this patient had a delayed diagnosis and manifested outside the newborn period. With mild symptoms secondary to her mural type VOGM, she lacked more severe sequelae. This makes prenatal diagnosis paramount. While ultrasound is a readily accessible, noninvasive option for imaging, angiography remains the gold standard. The advent of endovascular embolization has greatly reduced mortality in the pediatric population. This case demonstrates clinicians must maintain a high index of suspicion for vascular abnormalities with progressive macrocephaly in the absence of other symptoms, despite seemingly reassuring initial imaging, to abate outcomes with increased morbidity and/or mortality.
